# Exploring extracurricular clubs in high schools as third spaces for developing youth activism and critical engagement

**DOI:** 10.1111/jora.70034

**Published:** 2025-05-21

**Authors:** Tara Bartlett, Miri Yemini

**Affiliations:** ^1^ Mary Lou Fulton Teachers College Arizona State University Tempe Arizona USA; ^2^ Technion, The Israel Institute of Technology Haifa Israel

**Keywords:** activism, critical engagement, extracurricular clubs, K‐12 schools, third space, youth

## Abstract

Formal school spaces are often limited in preparing youth for critical engagement and activism due to standardized curricula, constrained resources, and politicized pressures. Nonformal learning environments, specifically extracurricular clubs, remain underexplored as spaces for youth activism. This study investigates how extracurricular clubs in high schools function as third spaces for learning about social justice issues and engaging in collective action. Using reflexive thematic analysis, we analyzed twelve discussion groups with 63 high school students involved in extracurricular clubs. Findings suggest that club participation fosters critical engagement, activism‐related knowledge and skills, and a justice‐oriented approach to local and global issues, with promise for enhanced global citizenship. However, specific conditions influence youth interest, retention, and action. Implications highlight the need for educational institutions to support third‐space opportunities for youth activism.

## INTRODUCTION

Amid concerns about global democratic backsliding, young people in various contexts—including the United States—have demonstrated significant potential to drive social change and challenge dominant power structures (Kubow et al., 2022; Teixeira, 2024; Wright & McLeod, 2023). Recent social movements such as Black Lives Matter, Fridays for Future, and March for Our Lives (see Appendix A for details) have increasingly been led and organized by youth. While many of these movements have gained international recognition, their impact is particularly pronounced within the U.S. context, where this study is focused.

Moreover, U.S.‐based youth are not insulated from global issues, partly because of increased access to technology and the effects of rising globalization. They engage with and are influenced by transnational movements, often drawing parallels between local injustices and global struggles. Many contemporary youth social movements transcend borders, emphasizing the interconnectedness of issues such as the climate crisis, human rights, and gender inequalities, while addressing injustices at both global and local levels (Karsgaard & Davidson, 2023; Kubow et al., 2022; Soler‐i‐Martí et al., 2020; Wilf & Wray‐Lake, 2024). These movements highlight the crucial role that young people play in efforts to create a more equitable, inclusive, and sustainable future.

While young people continue to spearhead powerful movements for social change, it is essential to acknowledge that the responsibility for tackling centuries of systemic issues cannot rest solely on their shoulders. Many of the challenges youth encounter today—ranging from the climate crisis to deep social and economic inequalities—are inherited difficulties intensified by the inaction or missteps of previous generations. As these systemic problems have become increasingly urgent in many parts of the world, young people often feel compelled to act, given the potential impact on their futures.

Simultaneously, the role of K‐12 schools and formal education institutions in providing opportunities for youth to cultivate critical engagement, knowledge, attitudes, and skills, as well as to participate in activism addressing social justice issues from local to global scales, has been limited (Aquarone, 2021; Kim & Kwon, 2023). In many countries, the political climate surrounding K‐12 curricula has discouraged schools from preparing young people with the knowledge, skills, and attitudes necessary to investigate and confront injustices within their communities and beyond (Groot et al., 2022; Groves & Stapnes, 2023; Levinson & Solomon, 2021). Furthermore, schools have faced backlash for integrating action civics and similar opportunities that have effectively fostered youth activist behaviors (Field, 2021).

In the U.S. context, the pressures of standardized testing and the enduring policy effects of the No Child Left Behind Act of 2001 have sidelined subjects that foster activism and utilize critical engagement pedagogies, particularly social studies and civics (Hubbard, 2013; Rock et al., 2006; Smith & Kovacs, 2011). The teaching of American exceptionalism has also become pervasive in schools' social studies and history curricula (An, 2022). This trend has coincided with the erasure of ethnic studies and the exclusion of diverse perspectives, including those of historically marginalized voices and experiences, from K‐12 social studies curricula (Shear et al., 2015; Tintiangco‐Cubales et al., 2015; Unidos U.S. & Johns Hopkins School of Education, 2023). Additionally, amid a backdrop of widespread, politically charged misinformation regarding the content and implications of critical race theory (CRT) in K‐12 schools, many U.S. states have implemented policies to restrict school curricula. Consequently, numerous educators have reported a chilling effect on their ability to teach about global issues and diverse perspectives (Kahne & Rogers, 2024).

Opportunities for youth to engage in democratic activities and address social justice issues through activism are also not equitable when considering access to time, resources, or support (Bauml et al., 2022; Mennes et al., 2023). For over a decade, research has demonstrated that white, more affluent students are likelier to experience high‐quality civic learning in K‐12 classrooms (Kahne & Middaugh, 2008; Lo, 2019). These inequities in access to civic learning opportunities underscore the necessity of exploring alternative avenues for youth engagement in educational institutions. Growing tensions within formal education systems have recently sparked interest in how learning beyond the classroom, particularly in school‐based third spaces like extracurricular clubs, can promote youth activism and critical engagement.

This study, therefore, seeks to deepen our understanding of whether and how young people are leveraging alternative spaces to pursue meaningful engagement and activism within schools by specifically addressing three research questions:

1. How are youth utilizing third spaces within schools?
2. What types of activism do youth prioritize in these third spaces?
3. According to youth perceptions, how might these third spaces facilitate the development of critical engagement and activism?

By exploring these questions, the study aims to contribute to the broader discourse on equitable civic education opportunities, youth activism, and critical engagement in the evolving yet intersectional landscape of education and democracy.

## GEN Z YOUTH ACTIVISM

Gen Z youth (born between 1997 and 2013) have experienced significant political and social turmoil during their prime years of sociopolitical development. The reasons are manifold. First, Gen Z youth have witnessed growing dysfunction in democracy amid global democratic backsliding (Cammaerts et al., 2014; Economist Intelligence, 2023; Foa et al., 2020). Second, Gen Z youth are aware of and consistently faced with profoundly entrenched and seemingly intractable problems, like climate change and human rights issues, a term coined by Head (2008, 2014) to describe policy problems with shifting needs and information, diverse actors affected and involved, fluctuating budgetary demands and ramifications, and relations to broader social and political issues. Third, Gen Z youth have cited concerns with their mental health and the concept of the common good, specifically a deterioration of relationships and a decreased sense of belonging, accelerated by the effects of the COVID‐19 pandemic (Bowler, 2020).

Nevertheless, studies demonstrate that young people have shown increased engagement in social issues and movements, taking up activism in new and nontraditional ways and charting their methods for political socialization (Earl et al., 2017; Teixeira, 2024). Gen Z youth have also embraced a hybrid approach to activism, where discourse, awareness building, and community organizing usually start online but translate into significant offline political participation, as seen in social movements like the Black Lives Matter movement, demands for reproductive freedoms following the overturning of Roe v. Wade and other women's rights, the accelerating climate crisis, the March for Our Lives movement, and demands for immigration reform via the DACA/Dreamers policies (Center for Information and Research on Civic Learning and Engagement [CIRCLE], 2018; Soler‐i‐Martí et al., 2020; Volpe, 2022; Wilf & Wray‐Lake, 2024).

Additionally, Gen Z is more inclusive of diverse identities and personal expression, more socially aware, partly due to growing up in the digital age, and more open to embracing diversity. In the United States, one in five Gen Z youth identifies as LGBTQ+ and is averse to binaries of gender, one in four identifies as Latina/o/x, and one in five has at least one immigrant parent, thereby recognizing and celebrating cross‐border cultures and identities (Parker & Igielnik, 2020). Gen Z youth have also been shown to be radically progressive and pro‐government (one study found that seven in ten youth believe the government should do more), although not necessarily pro‐democracy (ibid).

However, ageism and adultism continue to contribute to the democratic disenfranchisement of young people (Tisdall & Cuevas‐Parra, 2022). Gen Z youth often face the rhetoric of “wait‐citizenship,” a term coined by Bellino (2018), reflecting their struggle for structural inclusion and equality, specifically a lack of opportunities for structural inclusion that has contributed to young people's liminal positioning in society and their struggles to become social adults while actively seeking equality, democratic freedoms, and a sense of belonging. Some view Gen Z as too extreme, with a recent viral Los Angeles Times article citing that Gen Z youth embrace a “burn it all down” mentality due to their perceived low tolerance of intolerance by others who share different views (Guerrero, 2023). Furthermore, although Gen Z has been viewed as entitled and technology obsessed, research has found that young digital natives counter this stereotype with the notion that technology offers them convenience and efficiency in navigating everyday tasks, facilitates social connections and cohesion, and sparks creativity, visual motor skills, and problem‐solving abilities (Hackl, 2023; Haddock et al., 2022). Additionally, while youth‐adult partnerships in educational and political settings may often be presented in diverse ways, many reflect the bottom rungs of Hart's ladder of participation (1992), proving to be enacted as a range of unequal power behaviors from manipulation to tokenism. Since structured relations between young people and adults seldom allow space for authentic and proactive behaviors in young people, especially in formal settings, there is hope that less formal settings may provide an avenue for fostering youth activism and critical engagement.

## THIRD SPACES

Third space is a term used to describe an area that disrupts traditional structures and norms, allowing for new forms of engagement and agency. Bhabha (1994) refers to third spaces as sites for discovering or rediscovering participants' political and personal agency. In the school context, third spaces can be recognized as the educational spaces outside formal curricular mandates and classes that extend beyond the required school hours, such as extracurricular activities and clubs (Bamford & Moschini, 2024). These spaces are often staples in schools and can function similarly to in‐class attempts to foster civic engagement.

Extracurricular activities and student‐led clubs are frequently organized around a theme or specific topics and issues of student interest, providing an environment of “collective focus […] a shift in focus from individual to group” (Kirshner, 2007, p. 371). While many of these spaces are student‐led, they are usually supported by a faculty sponsor who must balance student agency with oversight responsibilities related to school policy and safety (Mitra, 2005). Like community youth organizing, student‐led meetings and initiatives through school clubs and extracurriculars mirror “social action processes in which youth operate as wielders and challengers of power in their local communities” (Christens & Dolan, 2011, p. 530). Indeed, many student clubs and extracurricular activities interact with contemporary issues and local community organizations through events, fundraisers, service learning opportunities, and other advocacy activities. Service learning is an everyday civic engagement activity that combines community volunteering alongside a critical analysis and learning opportunity of a particular social injustice (Billig et al., 2008). The literature on service learning has shown it to develop the propensity for community and civic engagement and self‐expression (Hart & Wandeler, 2018; Kahne et al., 2013; Zahedi et al., 2021). These lived experiences hold promise for facilitating fluid cultural exchanges and for “youth to develop […] their political socialization” (Earl et al., 2017, p. 3).

The research on K‐12 student clubs and extracurricular activities has shown positive outcomes across various areas. Studies have found extracurricular clubs successful in inducing voluntary associations, prompting deliberation about and action directed at a particular issue, and developing political efficacy and critical consciousness (Casanova, 2023; Levy, 2018; McFarland & Thomas, 2006; Park, 2016). Student participation in extracurricular activities and clubs has been linked to increased school attendance, socialization, and academic outcomes; improved school climates, and decreased risky behaviors (Brizuela, 2017; Feldman & Matjasko, 2005; Im et al., 2016; Lang, 2021). Additionally, preliminary research on teachers overseeing extra‐curricular activities has revealed that teachers build stronger relationships with students and increase their awareness and use of culturally relevant practices (Hensch, 2020).

However, the research on extracurricular clubs has also revealed challenges to equitable access, including funding for activities and fieldtrips, transportation after school, and time and resources provided to participating students and the adult sponsors, with inequities disproportionately affecting racially minoritized students and students from low SES backgrounds (Meier et al., 2018; Park & Kim, 2023; Snellman et al., 2015). Furthermore, limited research has been done on how student clubs and extracurricular activities, functioning as third spaces within K‐12 school systems, can equip young people with the necessary knowledge, skills, and attitudes to participate in activism and use tenets of critical engagement (Durand et al., 2025).

## CRITICAL ENGAGEMENT

We draw on Peterson et al.'s (2022) framework of critical engagement to examine how extracurricular clubs, as third spaces within K‐12 schools, support youth activism and critical engagement. Across the literature, critical engagement encompasses concepts such as critical consciousness, empowerment, reflection, action, and civic engagement—integrating an awareness of injustice with a belief in one's ability to take meaningful action (Casanova, 2023; Christens et al., 2023; Godfrey & Grayman, 2014; Pinedo et al., 2024; Wray‐Lake et al., 2024). Using the four dimensions within Peterson et al.'s framework, we explore the *context* of third spaces in developing youth activism, the *meaning and identity* these spaces provide for youth, the composition of *groups* involved in youth‐centered spaces, and the act and outcomes of *reflexivity* these third spaces offer.

The critical engagement of *context* focuses on a realistic understanding of the world's challenges and injustices. It moves beyond “simple political or moral messages,” rote memorization of static facts, and siloed analyses of complex issues (Peterson et al., 2022, p. 1096). Instead, context evokes a situated awareness, in which youth explore, interrogate, and strive to understand the historical, cultural, social, and political environments where events occur, alongside their own lived experiences and future‐oriented aspirations. Notably, youth who critically engage with perceptions of injustice and social inequality are more likely to be politically engaged in early adulthood (Diemer & Rapa, 2016; Rogers & Terriquez, 2013). Through opportunities for education, dialogue, and sharing lived experiences, youth can uncover the deep, intersectional dimensions of the issues they care about—such as climate change, systematic inequality, or human rights violations (Watts & Flanagan, 2007). This kind of contextual engagement supports youth in developing an analytical understanding of power structures and a sense of agency to act upon them.

The *meaning and identity* dimension of critical engagement centers on the positionality of youth and how they navigate modes and meanings of activism. Within school‐based third spaces, youth develop an understanding of personal and shared identities and values, shaping their motivations and actions. These spaces allow young people from diverse backgrounds to collectively understand their experiences and roles in the broader social world (Smith‐Appelson et al., 2023; Yosso, 2005). Because extracurricular clubs often coalesce around specific themes, they provide opportunities for youth to “foster a sense of common identity alongside the recognition of pluralism” (Peterson et al., 2022, p. 1098). Engaging with shared meaning and identity while addressing social issues can help youth build resilience, critical consciousness, and a proactive sense of purpose (Ginwright, 2010; Tuck & Yang, 2013). Moreover, negotiating complex social identities within group contexts contributes to youths' ability to work across lines of difference in solidarity and coalition.

Critical engagement with others in *groups* highlights the importance and dynamics of collective action while providing opportunities for civil discourse and trust‐building. The concept of groups in critical engagement is based on the understanding that meaningful change often requires collective effort and that working together with others can amplify impact. However, effective group functioning requires attention to identity, cohesion, diversity, and power structures that shape action outcomes (Ginwright, 2010). Within an extracurricular club setting, this may involve students engaging with one another, collaborating across clubs, working with adults on the campus, or building partnerships with community‐based organizations, while navigating potential adultism and tokenism (Mirra et al., 2013; Peterson et al., 2022). As part of a collective association, young people may become aware of how their sociopolitical identities and personal experiences shape their perspectives and actions within the group, fostering skills such as conflict management, critical thinking, and empathy (Romero & Cammarota, 2014; Watts & Hipolito‐delgado, 2015). Additionally, through shared meaning‐making and coalition‐building, youth can often navigate complex social identities and foster solidarity and community cultural wealth (Yosso, 2005).

Critical engagement in *reflexivity* is based on believing in the possibility of change, even amid systemic challenges. Concepts of critical hope (Freire, 2014), radical hope (Ginwright, 2010), and self‐efficacy (Bandura, 1997) are key for examining “one's own feelings, motivations, and actions and how these can and do influence actions and others” (Peterson et al., 2022, p. 1101). Reflexivity emphasizes that hope is essential for initiating and sustaining struggle while also recognizing difficulties ahead in striving for a more just future. Research has shown that hope can develop alongside young people's growing sense of agency and action in addressing injustice, suggesting a dynamic interplay between internal assets and external engagement over time (Kumar, 2024; Suzuki et al., 2023). In the context of extracurricular clubs, reflexivity and hope promote iterative learning, enabling youth to adapt and reflect on both the successes and limitations of their efforts. These concepts are particularly significant for youth activism, where young people often face intersectional oppression but remain determined to engage in reflexive spaces to cultivate resilience and transformative agency (Cammarota, 2007).

## SAMPLE AND METHODOLOGY

This research is part of a larger international project funded by the European Research Council, aimed at exploring and providing a comparative analysis of youth activism across six countries. While we were informed by analyzing data from other contexts, namely Australia, Italy, the United Kingdom, Poland, and Germany, these findings are specific to one southwest U.S. locality and focus on exploring how youth navigate third spaces for activism and engagement within a distinct national context. Therefore, we acknowledge generalizability as a limitation.

For this study, 63 high school‐aged youth from six rural and urban low‐socioeconomic status (SES) public schools were recruited to participate in one‐time, semi‐structured group discussions. We intentionally sought to engage youth from diverse geographic and social contexts to ensure a variety of perspectives on activism and critical engagement within school‐based third spaces. The study was approved by Arizona State University's Internal Review Board and leadership within each district, and parent consent and youth assent were obtained before the 12 discussion groups took place. We did not collect or report detailed demographic percentages due to IRB guidance and state law restricting inquiries about student gender identity, sexual orientation, and other personal characteristics (ARS 15–117, 2022). Moreover, there has been a chilling effect across school districts since the Arizona Superintendent of Public Instruction set up a hotline for complaints about teachers discussing “inappropriate” topics of race, gender, and sexuality (Kunichoff, 2023). This has recently been followed with threats of withholding funding from K‐12 public schools if they do not acknowledge that they will follow federal civil rights law and avoid the use of Diversity, Equity and Inclusion terms and programming (Arizona Department of Education, 2025). However, we can share aggregate demographic information. The youth participants ranged in age from 15 to 19 years old and were primarily nonwhite, including students who identified as Latino/a/x, Black, and Asian. We can also confirm that students self‐identified across a spectrum of gender identities, including male, female, nonbinary or queer, and transgender.

Recruitment focused on extracurricular clubs centered on social, political, or environmental issues, student‐led service learning, and community engagement activities. These clubs included She's the First Club (focused on women's and girls’ empowerment), Model United Nations (Model UN), Gay‐Straight Alliance (GSA), Green Climate Club, National Honor Society (civic and service leadership), and We the People (focused on civics, law, and constitutional issues). The diverse thematic focus of these clubs provided a rich context for exploring how youth engage in activism and critical reflection within school‐based third spaces.

Once the study received IRB approval, the first author contacted adult sponsors for these clubs within local schools to arrange dates and times to visit and conduct the group discussions. In arranging the group discussions, the first author drew upon collegial relationships with many of the adult sponsors or was introduced to the adult sponsor via a shared colleague. Parent consent and youth assent were obtained before the youth participated in the group discussion. Participating youth were also briefed on the larger scale of this project as a cross‐country comparison of youth critical engagement and activism.

The first author conducted the twelve semi‐structured discussion groups from November 2023 through May 2024 on school campuses or via Zoom, each involving a unique group of youth. Discussion groups ranged from 60 to 90 min with three to six participants, depending on club member availability. The discussions followed a pre‐specified protocol of guiding questions organized in three parts: (1) how the youth engaged in social justice‐oriented topics and activities within these third spaces, (2) which activism and advocacy methods were used and prioritized, and (3) to what extent their experiences in these third spaces fostered activist behaviors and critical engagement. The first author posed the prompts and questions from the protocol for the youth to discuss with their peers. While a core set of questions was used across all sessions to maintain consistency, the semi‐structured format allowed for organic evolution of discussion topics, particularly as youth introduced new insights or elaborated on shared and lived experiences (Morgan et al., 1998). This approach enabled flexibility while ensuring that key themes were consistently explored across all groups. The format encouraged peer‐to‐peer reflection and collective meaning‐making, aligned with the study's focus on third spaces and youth agency.

The audio of the discussion groups was recorded and transcribed for analysis. Transcriptions were analyzed using reflexive thematic analysis (Braun & Clarke, 2021), facilitating identifying and comparing emerging themes across the data. This method is an iterative process that involves interpreting data, in this case, individual statements or phrases across the discussion groups for specific questions to establish categories and themes. After conducting the discussion groups and becoming familiar with the data, the first author conducted an inductive coding process, closely reading each transcript and generating initial codes based on recurring patterns, language, and ideas expressed by participants. These codes were sorted into themes and refined iteratively through discussion with the second author as intercoder agreement, with attention to semantic and latent themes. As new insights emerged, categories were refined and (re)defined to reflect the data accurately. The six‐phase trustworthiness criteria from Nowell et al. (2017) were also employed to ensure the rigor and reliability of the analysis. MAXQDA (VERBI Software, 2022), a qualitative data analysis software, assisted with the coding process. MAXQDA assisted with the management and organization of the data, allowing for both the inductive and deductive coding cycles to expand and contract during analysis as new categories or themes emerged. This method ensured a systematic approach to analyzing the discussion groups while preserving the flexibility to explore unanticipated insights. The final themes reflect the shared and diverse perspectives of the youth participants and are presented in alignment with the study's three guiding research questions.

## RESULTS

The findings from the twelve discussion groups revealed consistent themes related to youth engagement in extracurricular clubs as third spaces for activism and critical reflection. Results are presented in relation to the study's three guiding research questions.

### Research question 1: How are youth utilizing third spaces within schools?

A significant theme shared across the discussion groups was the use of the clubs as third spaces to foster student choice and pursue student‐led initiatives that were otherwise invisible in schools. These initiatives stemmed from student interests in issues often omitted or avoided in formal curricula, including climate change, LGBTQ+ rights, human rights, and reproductive rights. Many students cited a need for the freedom to explore these topics beyond the mainstream classroom, with one student in a We the People Club highlighting, “Honestly, learning about human rights, I think that at least like in our education system, we definitely don't learn enough about it” and a Model UN student sharing, “There's like so many just useless classes out there, but the important stuff like this just doesn't exist.” Similarly, students within a Green Climate Club conceded that “Climate‐wise, I haven't gotten any formal information from classes” and “I haven't learned much about global issues, so I've had to do a bunch of research outside of actual public school, which is unfortunate.”

The students also discussed using these third spaces to address perceived gaps in their political education and to advocate for club recognition when dealing with controversial or politicized topics. For instance, members in a GSA club shared their efforts to “create this movement for like school rights and LGBT and, like, all this stuff. And, like, we should be able to have clubs that are supporting political stuff as an accepted extracurricular for the school.” One student in a GSA club shared, “I feel like [the teachers] are scared to get too controversial. Yeah, like they don't know if they're allowed to, especially like in recently, in like Florida, where the teachers can get fired or arrested for saying certain words.”

Similarly, students in a She's the First club discussed how their mainstream classes shied away from any conversation around politically charged issues, specifically citing abortion and women's rights. One student in this club stated, “I can't recall any information that I've learned about women's rights in class, especially about Roe v. Wade. When that was happening, teachers were really like, not bringing it up at all because people had differing opinions.”

However, considering the learning constraints, students advocated for the expansion and support of these third spaces, with one student in the Model UN club expressing,

Educational institutions [should] give students outlets where they can express and things other than obviously school, you know, after school activities, events. Those things are so important for students that are, you know, constantly going through stress, especially coming into somewhere like high school and leaving from middle school, having outlets where they can kind of find their passions, find what works for them, even like discover traits about themselves, leadership roles or critical thinking.

A second theme apparent across the discussion groups concerns how youth used these third spaces as sites to counteract the concept of ‘wait citizenship.’ A few students described feeling as though young people were often made to feel as if they are in a waiting or holding pattern until they are of legal age to be able to engage as citizens, with one We the People student sharing having been told, “‘There's not much you can do for now.’ Like, we're told we can't vote, we can't pay taxes, we can't really make big decisions. We just have to wait because there's only a little bit we can do.” Another student from Model UN shared this sentiment: “I feel like a lot of times everyone always thinks like, ‘Oh, teenagers are so immature. We shouldn't talk about these big issues in schools. They're not ready for it.’ But then, again, we can drive, and a lot of us can vote.” Conversely, other students felt as though their semi‐autonomous decision‐making and leadership opportunities within the club debunked the idea of wait‐citizenship, with an NHS student expressing, “Because even at 17, you could still do things […] I think it's just so much about the fact that you're dedicated and want to continue to grow the community.”

### Research question 2: What types of activism do youth prioritize in these third spaces?

Youth participating in the third space clubs engaged in a range of activism activities and behaviors, including peer education, service learning, protests, and coalition‐building. One prominent activity students engaged in was taking on the role of an information activator among their peers. In this role, students shared knowledge and lived experiences on social issues, reclaiming the role of teachers and divulging stories and ideas driven by personal interest and choice. As one student in a We the People club explained, “I feel like educating others is something we can do or just like really being aware and thinking about awareness because it's like educating yourself and educating others.”

Overall, students believed that sharing information on specific issues was dually crucial for their peers within the club and those outside of these third spaces, with a student in a Model UN Club emphasizing the need to “getting involved with your community and then presenting or talking to people about it, like kind of informing people because a lot of people may be ignorant or they just may not be educated on these subjects.” Many of the students also cited wanting to share about specific topics that were personal to them or for which they advocated for change, with one student in a We the People club sharing, “I talk a lot about prison reform because like, for example, when I was writing about it was like in Texas, I believe, where they found out that a lot of the prisoners in there were being mistreated.”

Service learning emerged as a common type of activism students took up in these third spaces, particularly in clubs like She's the First Club and NHS. Students in a She's the First Club discussed how they “organized and fundraised money for menstrual products, and we all created packs to give to people who were displaced and don't have access to them.” Another student in a National Honor Society organization shared a belief “that every child should have access to services, like having access to an amazing education” and then described how they hosted activities centered around educational access and mentorship within their school and broader community. Although volunteering is sometimes considered a strategy for social reproduction and résumé enhancement for more privileged students (Dougherty et al., 2018), in this case, the service learning activities were driven by students from historically marginalized populations.

Students also engaged in direct advocacy, including school walkouts, attending school board meetings, and contacting local representatives. One student in a Green Climate Club outlined the importance of using one's voice in a myriad of ways to advocate and participate in a range of activist behaviors:

I think we need to be using our voice in as many ways as possible. Like, if you don't want to be the type of person who goes up and speaks, maybe like writing things, creating graphics, doing research, or what I mentioned earlier, like attending a strike or like, literally speaking, to the politicians who are in their rooms watching us. I just think we need to get other people motivated to like know your voice does matter.

Additional students in a Green Climate Club shared efforts of advocacy and partnership as part of their activities by describing how they “recently just met with the school's sustainability board […] to start implementing projects to work with the lower school and middle school to have them get involved in some of our projects, too” and that they were “contacting representatives and building a coalition of businesses, the other stakeholders, and legislators for Clean Climate Energy to pass in Arizona, and also put in our AZ Youth Climate Coalition strike demands.” Other behaviors included the GSA club organizing a walkout from school and attending the school district's governing board meetings to demand equitable treatment of LGBTQ+ students and, specifically, access to gender‐neutral bathrooms for trans students to feel safe and comfortable on campus.

### Research question 3: According to youth perceptions, how may third spaces facilitate the development of critical engagement and activism?

Youth reported that third space clubs fostered awareness of local and global issues and created a shared purpose. The youth discussed the concepts of *meaning and identity* when describing a shared understanding of issues on the local and global scales. Students cited learning about topics such as the wars in Gaza and Ukraine, LGBTQ+ rights, and climate change, often drawing connections between global events and local impacts. One GSA student remarked, “I think a common theme I learned is how human rights are just so interconnected to a lot of the other larger issues that are human‐caused,” while a We the People student cited, “When talking about political polarization, the worst part is, it's been like that for so many years that you can't really just stop it, or fix it, but I mean, we can certainly try.”

Students also described the composition of the clubs as influencing their engagement, noting the value of diverse lived experiences in shaping discussions, collective identity, and approaches to activism. In terms of the composition, many youth cited a realization of the power of diverse lived experiences when discussing issues and participating in activities to spark change within their communities concerning these issues, particularly in light of power dynamics. A student in the National Honor Society club described this sentiment by sharing, “I feel like the best way to approach it [the club] and to like make it a safe space is with values you have because like, everyone has different values.” These themes were captured by a student in a She's the First Club who stated, “A lot of people say one person can make a difference. But I feel like in this situation, you really can't because we all have to work together because it's mostly against the most powerful people, and we the people have the power to make a change in this situation,” and a student in a Green Climate Club explaining that “Once people start to learn about things that they have in common with each other, they start to then care for each other, and that helps solve the issues.” Students frequently mentioned that the composition of the student groups in these spaces was demographically diverse, yet they shared that the club fostered a space for curiosity and understanding while also sharing similar viewpoints and values concerning particular issues.

Finally, students spoke of reflexivity expressed as a sense of agency and critical hope in their ability to create change. One student exemplified the ideas of reflexivity and critical hope by sharing, “It's like inspiring because I know that like now, I have an opportunity to create change and do what I want because I see a problem and now I’m more incentivized to fix it.” A student in a Green Climate Club described the powerful potential of this approach: “I think one of the things that has really helped me understand the impact of climate change on marginalized communities more is meeting with people who are suffering the most. Like meeting face to face, because just hearing about it and reading about it is different than talking to someone and learning their story and their personal understanding of the issue.” Furthermore, the development of self‐efficacy was described by a student in a GSA club as “[it] really opens students' eyes to like the many inequalities that we have and could possibly inspire change.”

## DISCUSSION

This study explored how high school youth utilize extracurricular clubs as third spaces to foster activism and critical engagement. The findings highlight both the opportunities and constraints youth experience in these spaces. In this section, we interpret the key themes and findings through the research questions and the lens of Peterson et al.'s (2022) critical engagement theory (Table 1). Findings support acknowledging a broader context of youth activism, opportunities for youth sociopolitical development in schools, and the relevancy of Global Citizenship Education (GCE) through the students’ experiences in these third spaces.

**TABLE 1:** Summary of research questions, key findings, and alignment with Peterson et al.'s (2022) critical engagement framework.

Research question	Key themes/Findings	Relevant component(s) of Peterson et al.'s (2022) framework
1. How are youth utilizing third spaces within schools?	Exploration of topics omitted in formal curricula (e.g., climate change, LGBTQ+ and women's rights) Countering “wait‐citizenship” and adultism	Context Meaning and Identity Group Engagement
2. What types of activism do youth prioritize in these third spaces?	Peer education and awareness‐raising Service learning and community engagement Direct advocacy and coalition‐building	Group Engagement Reflexivity
3. How might third spaces facilitate critical engagement and activism?	Awareness of global–local issues Self‐efficacy and critical hope Shared identity and resilience Reflexive storytelling and personal connection	Context Meaning and Identity Reflexivity Group Engagement

Youth participants exhibited varied understandings of the term “activism” but showed similar actions across club activities like coalition‐building, service learning, and advocacy. These engagement activities enabled students to amplify their voices, navigate complex decision‐making structures, and recognize the importance of coalition‐building to achieve their goals related to local and global issues. Their activism reflects a broader trend of youth‐led movements gaining prominence at local, national, and global levels as young people increasingly acknowledge their agency and responsibility to advocate for social change. While students came from diverse backgrounds, they found that these differences strengthened them and nurtured a sense of solidarity and common purpose when addressing shared concerns in their clubs.

In analyzing how the third space clubs facilitated the development of critical engagement among youth, several themes emerged in line with the Peterson et al. (2022) critical engagement framework. For one, the *context* of these third spaces fostered a healthy curiosity and awareness of social justice and local to global issues. Students cited learning about issues within their school community, state, and nation, as well as the world broadly, including issues such as the wars in Gaza and Ukraine, female genital mutilation (FGM), LGBTQ+ rights, reproductive rights, climate change impacting delicate ecosystems such as coral reefs, and the civil war in Sudan, among others.

This contextual engagement also reflected youth awareness of power dynamics, especially when navigating politically charged or controversial issues within their schools. Youth emphasized how the clubs filled gaps left by formal curricula, playing significant roles in their knowledge formation and motivation to act. Outside of a few specific elective courses, formal learning spaces in schools seemed less effective in inspiring youth to enact change within their communities. Students expressed a shared desire and understanding of using clubs to explore, discuss, and take action on issues that were intentionally avoided by their teachers, including issues of LGBTQ+ rights, specifically trans student rights, sex education, women's rights, immigration, human rights, the climate crisis, and racism, and were keenly aware of current educational politics barring teachers from discussing specific topics in the classroom. Again, these issues were not heavily explored in their mainstream classes, so students used clubs to self‐educate and spread awareness about such issues among each other within these spaces and with peers outside of these spaces.

The resilient determination of youth working together in *groups* to effect change speaks to their understanding of power and agency. Many students cited a newfound awareness of their collective power when addressing social issues and participating in activities to spark change within their communities. For example, the students’ efforts in contacting representatives and engaging with diverse stakeholders suggest an awareness of the power dynamics at play and the necessity of building a broad‐based coalition of support to achieve their goals. The realized learning of power dynamics and structures among groups was also especially apparent when students wanted to organize an event with peers or advocate for change within their school community. This finding aligns with the literature on student voice, autonomy, and motivation, particularly as articulated in self‐determination theory (Conner et al., 2024; Evans & Boucher, 2015).

The dimension of *meaning and identity* was also central to youth engagement. Clubs provided a space for young people to explore and affirm personal and shared values, develop resilience, and foster a proactive stance toward social justice. Youth cited their diverse experiences as strengths and emphasized unity around shared concerns, demonstrating a clear understanding of power and agency, particularly in utilizing these third spaces to combat and address issues of wait‐citizenship and adultism. With the concept of wait‐citizenship heavily influenced by beliefs and practices of adultism, students expressed frustration with school leaders and other personnel outside of the club sponsors who did not trust them to engage in specific topics or decision‐making processes. Through the experiences and opportunities offered in the clubs, along with the club sponsors supporting best practices of youth‐adult partnerships, students could engage in impactful behaviors and activities to enact change within their community. These experiences resonate with theories of community cultural wealth (Yosso, 2005) and youth organizing frameworks that prioritize collective identity and solidarity (Ginwright, 2010).

Youth also engaged in *reflexivity*, expressed through critical hope and self‐efficacy. Many students employed a self‐storying strategy while reflecting on their reflexivity, critically examining their roles within the broader social movements and issues highlighted, including personal narratives of how experiences from their childhood, interactions with family and friends, and community connections had shaped their worldviews and areas of interest. Freire's (2014) concept of critical hope as essential for sustaining activism and Duncan‐Andrade's (2009) emphasis on hope as resistance are evident in how youth utilized reflection and personal storytelling to ground and sustain their engagement.

Youth participants frequently cited learning about global issues—such as climate change, human rights, and reproductive rights—within extracurricular clubs, noting the absence of such topics in formal curricula. Given the political and structural barriers to implementing Global Citizenship Education (GCE) in U.S. classrooms, school‐based third spaces like extracurricular clubs may serve as alternative avenues for youth to engage with global issues and develop competencies aligned with GCE objectives. Indeed, knowledge of global issues is particularly important given that universal access to GCE is a crucial target of the United Nations (UN) Sustainable Development Goals, to be achieved by 2030. The SDG4 mission is to “Ensure inclusive and equitable quality education and promote lifelong learning opportunities for all.”

Embedded within the extracurricular context, GCE can transform education systems and empower learners to build “more peaceful, tolerant, inclusive, secure, and sustainable societies.” However, we must recognize the difficulties in navigating GCE within US schools: historically, the US education system has faced challenges in promoting GCE due to political isolationism, a focus on standardized testing, and a lack of teacher professional development in GCE (Rapoport, 2015; Rapoport & Demir, 2022). Decentralized (state) governance and a lack of national (federal) teaching standards further complicate the integration of global competencies into the curriculum. Pointedly, little research has been done to explore how K‐12 standards across the United States are appropriating GCE and, if so, how and to what extent GCE is incorporated within each state's K‐12 teaching standards or locally controlled school governing board's curricular decisions (Rapoport, 2009). According to the students in the discussion groups, their most significant opportunities to learn about global issues exist in extracurricular clubs.

The opportunities provided by K‐12 extracurricular club third spaces have proven effective in encouraging students to explore and pursue their interests, reflect on content and context, and critically examine how various issues impact themselves and others. These third‐space clubs provide young people with opportunities to critically navigate and engage with the complexities of identity and power in ways often unavailable in traditional educational environments. Despite their significance, third‐space clubs frequently lack institutional support and are underfunded when compared to formal curricular programs. Nevertheless, these spaces continue to evolve in response to the interests and needs of youth. They provide vital opportunities for young people to navigate identity, power, and activism in ways not typically accessible in traditional education settings. In summary, third spaces can prepare the next generation of civic leaders by embedding equity, resilience, and collaborative action at the core of youth activism and critical engagement.

## LIMITATIONS AND RECOMMENDATIONS

While this study reveals critical insights into how youth engage in activism and foster critical engagement within third spaces, a few limitations should be noted. First, the data collected is context‐specific and not entirely generalizable since all participating youth attended public high schools and took part in themed extracurricular clubs in a single locality in the southwest United States. Indeed, the experiences of youth in different educational contexts may vary depending on curriculum oversight, access to extracurricular clubs, teacher autonomy, and the intrinsic culture of a school community. Although these findings are rooted in a specific U.S. context, they provide insights into how globally relevant movements and issues are negotiated locally, particularly within school‐based third spaces.

Second, the sample size (*n* = 63 participants, 12 discussion groups) is adequate for achieving empirical saturation (Hennink & Kaiser, 2022). However, the perspectives and experiences of the participating high school youth are far from monolithic, particularly outside of the specific clubs that act as third spaces of communal gatherings focused on shared interests and values. Similarly, like the contextual limitation, additional discussion groups representing a wider range of views and lived experiences could offer deeper insights into how young people utilize third spaces and the issues they are passionate about.

Findings from this study provide insight into how schools and other educational institutions can create more spaces to foster activism and critical engagement among youth. Much of the existing literature on youth activism and efforts to develop critical consciousness, specifically among school‐age youth, focuses on partnerships and engagement within community organizations. Moreover, while third spaces within schools can undoubtedly serve as access and development points, efforts to offer different topics and engagement methods and equitable access must be expanded. These efforts may include curricular‐embedded experiences and partnerships with different community‐based organizations, including issue‐focused, advocacy, or faith‐based organizations.

One point raised among the students across different discussion groups and likewise suggested as an additional area to explore in Peterson et al.'s (2022) critical engagement framework is the interconnectivity of youth in schools to civil society organizations or “community‐based educational work” where “teachers and non‐teachers could work together” (Peterson et al., 2022, p. 1101). In practice, this may include the extra‐curricular clubs partnering with different community organizations on service learning activities or building advocacy networks. However, it could also include teachers brokering connections and relationships with everyday people in the surrounding community affected by or working on similar issues as the club. Additionally, schools may consider cross‐generational youth‐adult or peer‐to‐peer mentorship opportunities and online spaces to explore and connect with other individuals and groups working to address global issues. Moreover, simple logistics such as providing space on a school campus and reliable transportation if the club takes place after school hours should be considered.

We recommend two areas for future research. First, while this study focused on school‐based third spaces, future research might explore how digital platforms—central to the digital democratization of knowledge and mobilization—serve as parallel or intersecting third spaces. Given their growing relevance in young people's lives, online spaces may play a critical role in shaping youth activism and critical engagement. Second, future research could focus on the role of teacher sponsors within these extracurricular third spaces. While organizing our discussion groups, we interacted with the clubs' teacher sponsors. Sometimes, they were present in the room—either working quietly or listening during the discussions. However, these sponsors did not participate directly in the discussion groups. This raises questions such as: (1) To what extent do teacher sponsors support the educational and advocacy goals identified by student members in these third spaces? Moreover, (2) Does alignment between the sponsor's values and the students' interests impact the quality of learning about social justice issues, the effectiveness of advocacy and activism efforts, and the overall perceived success of the club's initiatives? Previous research has highlighted creative ways educators have integrated youth activism and critical consciousness into their mainstream teaching (Cho, 2018). However, such approaches remain the exception rather than the norm, particularly given the current social and political pressures on public educational institutions. Therefore, we are interested in how educators support and cultivate these learning opportunities outside formal classroom structures.

## CONCLUSION AND SIGNIFICANCE

This study emphasizes the need to examine how third spaces within schools foster critical engagement, resulting in youth taking up activist behaviors outside and beyond formal schooling spaces. Discussion groups with high school students involved in third space clubs explored how Gen Z youth understand and adopt activism, develop an awareness and understanding of global issues, which methods are used to engage with issues they care about, and the role of these third space clubs in fostering critical engagement. Findings reveal that extracurricular third space clubs support engagement in activism by concentrating on issues not explored or discussed in their mainstream classes or formal education spaces. However, many young people still have diverse understandings of activism, global issues, and how to enact different methods of learning about and addressing these issues.

Markers of critical engagement are fostered in these third spaces, alongside experiences in advocacy, service learning, and partnering with local organizations to influence key decision‐makers, policies, and practices within their school and local communities. Engagement in these spaces developed youths' understanding of social justice and local and global issues and significantly enhanced activism and leadership skills. Further, youths cited the limited impact of formal education on their modes of activism and the development of critical hope and consciousness, emphasizing the importance of these spaces.

Overall, third spaces in K‐12 educational institutions hold promise in fostering student voices, delivering inclusive learning content, and providing opportunities to engage in civil discourse. This study's results can inform schools and educational institutions about the power and potential of third spaces to develop youth activism and critical engagement for long‐term civic engagement and more equitable communities. As youth continue to face social, political, and environmental challenges, educational institutions must recognize and support the transformative potential of these third spaces in cultivating a more just and participatory democracy.

## FUNDING INFORMATION

The study received funding from the European Research Council (Grant Agreement # 101082917). Funding compensated the authors for their time collecting and analyzing the data and writing the findings for this study.

## CONFLICT OF INTEREST STATEMENT

There are no conflicts of interest to disclose.

## CONSENT STATEMENT

Parent consent and minor participant assent were obtained for participation in this study.

## Data Availability

Data are available upon request from the authors.

## APPENDIX A

*Black Lives Matter* is a social and political movement advocating against systemic racism and violence toward Black individuals, especially in response to police brutality.

*Fridays for Future* is an international youth movement demanding climate action, initiated by Swedish activist Greta Thunberg, in which students strike from school to protest political inaction on the climate crisis.

*March for Our Lives* is a youth‐led movement advocating for stronger gun control laws in the United States, initiated in response to the 2018 school shooting in Parkland, Florida.

*Roe v. Wade* was the landmark 1973 U.S. Supreme Court decision that federally protected the right to abortion; it was overturned in 2022 by *Dobbs v. Jackson Women's Health Organization*, leading to significant restrictions or bans on abortion in many states.

*DACA* (Deferred Action for Childhood Arrivals) is a U.S. immigration policy that offers temporary protection from deportation for eligible undocumented youth, often referred to as *Dreamers*, who were brought to the country as children.
